# Inhibitory Effect of Imperatorin on the Pharmacokinetics of Diazepam In Vitro and In Vivo

**DOI:** 10.3389/fphar.2020.01079

**Published:** 2020-09-16

**Authors:** Yunfang Zhou, Deru Meng, Feifei Chen, Zhengping Wu, Binglan Wang, Shuanghu Wang, Peiwu Geng, Dapeng Dai, Quan Zhou, Weiwen Qiu

**Affiliations:** ^1^ The Laboratory of Clinical Pharmacy, The Sixth Affiliated Hospital of Wenzhou Medical University, The People’s Hospital of Lishui, Lishui, China; ^2^ College of Chemistry and Bioengineering, Yichun University, Yichun, China; ^3^ Department of Neurology, The People’s Hospital of Longquan, Longquan, China; ^4^ The Key laboratory of Geriatrics, Beijing Institute of Geriatrics, Beijing Hospital, National Center of Gerontology, National Health Commission, Institute of Geriatric Medicine, Chinese Academy of Medical Sciences, Beijing, China; ^5^ Department of Neurology, Lishui Hospital of Traditional Chinese Medicine, Lishui, China

**Keywords:** imperatorin, diazepam, drug-drug interaction, pharmacokinetics, P450

## Abstract

**Background:**

Diazepam is a benzodiazepine drug used to treat anxiety, insomnia, and muscle spasms. Imperatorin is a phytochemical isolated from medicinal plants and is widely used in herbal medicine. The aim of this study was to investigate the interactions between imperatorin and diazepam *in vitro* and *in vivo* and to provide evidence-based guidance for the safe clinical use of the drug.

**Methods:**

In vitro inhibition of imperatorin was assessed by incubating rat liver microsomes with diazepam to determine IC_50_ values and the type of inhibition. For *in vivo* assessment, six rats were pretreated with 50 mg/kg imperatorin for two weeks, six were administered saline, and a single dose of 10 mg/kg diazepam was administered orally to both groups 30 min after the administration of imperatorin.

**Results:**

Imperatorin inhibited the *in vitro* metabolism of diazepam *via* the competitive mechanism of CYP450. The IC_50_ values of imperatorin to nordazepam and temazepam were 1.54 μM and 1.80 μM, respectively. The inhibitory constant values for temazepam and nordazepam were 1.24 μM and 1.29 μM, respectively. Long-term administration of imperatorin significantly increased the AUC_(0-12h)_, AUC_(0-∞)_, and Cmax of diazepam, while Vz/F and CLz/F were decreased significantly (*P* < 0.05). In turn, the AUC_(0-12h)_, AUC_(0-∞)_, and Cmax of nordazepam and temazepam decreased significantly, and Vz/F and CLz/F increased significantly (*P* < 0.05).

**Conclusions:**

This study demonstrates that imperatorin inhibits the metabolism of diazepam both *in vitro* and *in vivo*. These results indicated that more attention should be paid when taking diazepam together with food or herbs containing IMP, although further investigation is still needed.

## Introduction

Imperatorin (IMP), a linear furocoumarin compound, is widely found in the roots of the medicinal plants *Cynanchum otophyllum* Schneid, *Angelica sinensis*, and *Angelica dahurica* (García-Argµez et al., 2000; Zhang et al., 2010; Badziul et al., 2014; Morris et al., 2018). IMP is an important active ingredient of various widely used traditional Chinese medicine preparations such as Huoxiang Zhengqi Shui and Yuanhu Zhitong tablets, with anti-inflammatory, analgesic, anti-tumor, anticonvulsant, and anticoagulant pharmacological effects (Stavri and Gibbons, 2005; Luszczki et al., 2007; Rosselli et al., 2007; Zhang et al., 2009). IMP also has good antibacterial activity and different degrees of inhibition on *Escherichia coli*, degenerative bacteria, *Staphylococcus aureus*, and *Salmonella typhimurium* (Tsassi et al., 2010). In addition, it can also inhibit the infection of T cells and HeLa cells by human immunodeficiency virus type 1 (HIV-1), vesicular stomatitis virus pseudotype, and glycoprotein gp160-enveloped (Sancho et al., 2004). In a study on the effect of extract of white peony on the thoracic arteries of mice, the degree of vasodilation was observed to decrease in a dose-dependent manner, and the main active contributor to this effect was determined to be IMP (Nie et al., 2009). The multiple pharmacological activities of IMP indicate its great potential as a clinical drug.

Many studies have reported that IMP is mainly metabolized in the liver, and only a small amount is excreted through the kidney in a prototype form, which suggests that IMP is prone to biotransformation *in vivo* (Liu et al., 2011). Previous studies have suggested that IMP might play a major role in the pharmacodynamics of coumarins and their interaction with cytochrome (CYP) enzymes (Nie et al., 2009). The interaction between IMP, the active ingredient in couramins, and CYP enzymes is complex. Furanocoumarins have been reported to inhibit cytochrome 450 (CYP450) enzyme activity (Feng et al., 2018). In human liver microsomes, IMP and IsoIMP have different degrees of inhibition on six CYP isozymes, with potent inhibition of CYP1A2 and CYP2B6, moderate inhibition of CYP2C19, and weak inhibition of CYP2C9, CYP2D6, and CYP3A4. In rat liver microsomes (RLMs), IMP moderately inhibits CYP1A2 and CYP2B6, and both IMP and IsoIMP are weak inhibitors of CYP2D2 and CYP3A1/2 (Kimura et al., 2010; Kang et al., 2011). The results of these studies show that IMP has a wide inhibitory effect on human liver CYP enzymes, and, in clinical settings, attention should be paid to the interaction caused by CYP enzyme inhibition.

**Figure 1:**
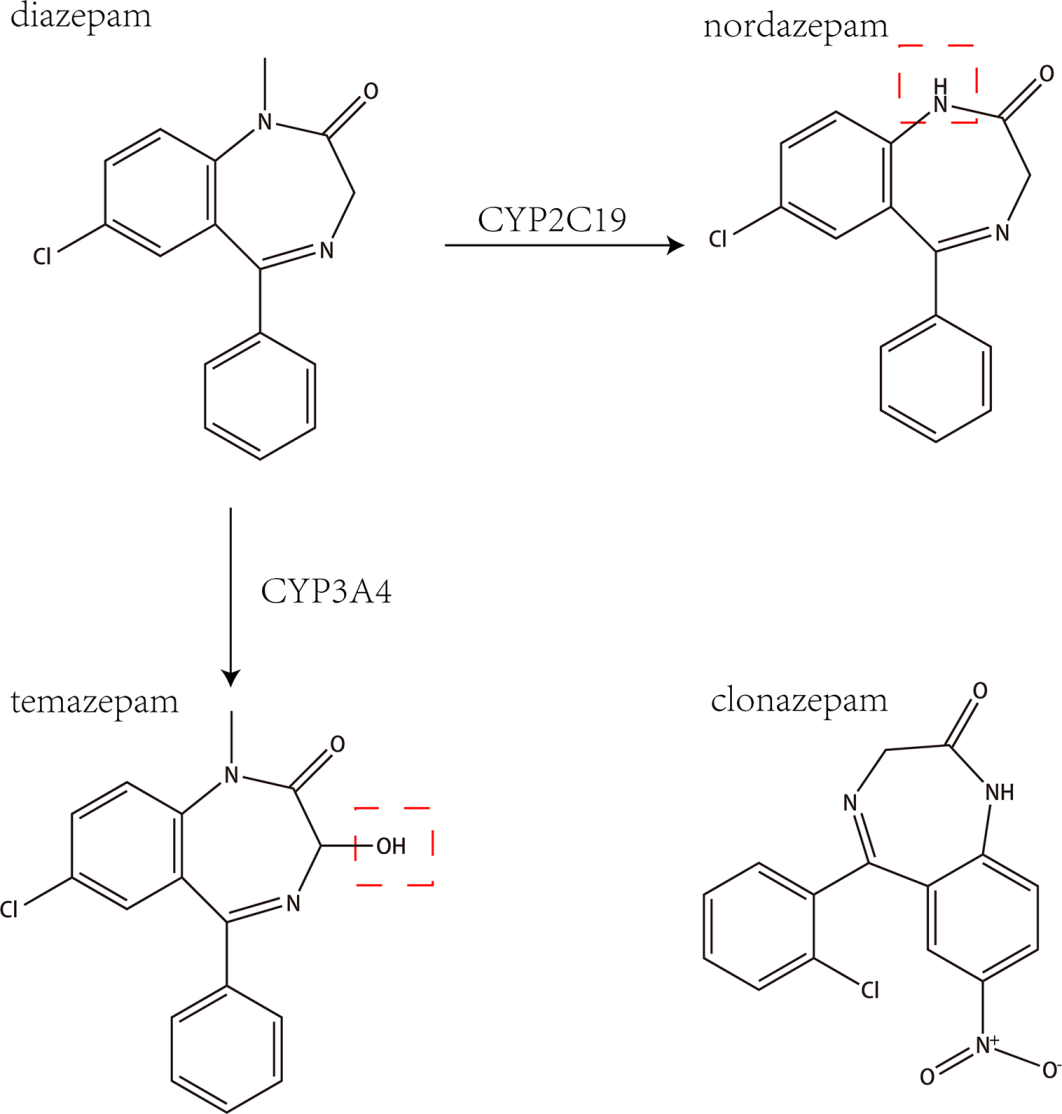
The chemical structures and mass spectra of diazepam, nordazepam, temazepam, and clonazepam (internal standard).

Diazepam is a benzodiazepine drug with a variety of clinical uses, including the treatment of anxiety, insomnia, muscle spasms, seizures, and other symptoms. Diazepam has a variety of effects on CYP450 enzyme activity, mainly through the metabolism of CYP2C19 and CYP3A4 into the main active metabolites nordazepam and temazepam, as shown in **Figure 1** (Sakai and Ishizuka, 2009). Studies have shown that diazepam inhibits the activities of CYP2B6 and CYP1A2 in rats (Andresen et al., 2013).

The CYP450 enzyme system is the most important drug metabolizing system in the liver and is rich with CYP subtypes (Thakur et al., 2007). Human and mouse CYP450 enzymes have a high degree of homology, with very similar metabolic functions. CYP450 enzymes mainly consist of CYP1A2 (4% of total CYP drug metabolism), CYP2C9 (10%), CYP2C19 (2%), CYP2D6 (30%), CYP2E1 (2%), and CYP3A4 (50%). These enzymes account for more than 75% of all CYP450 enzyme levels in the liver and participate in the metabolism of more than 90% of clinical drugs (Ravyn et al., 2013).

Compared with numerous pharmacodynamic studies, studies on the metabolism and pharmacokinetics of IMP and diazepam in rats *in vitro* and *in vivo* have been unsystematic. This lack of systematic evaluation necessitates further studies focusing on the CYP inhibitory activity of IMP on diazepam. In determining IMP and diazepam levels, ultra-performance liquid chromatography-tandem quadrupole mass spectrometry (UPLC-MS/MS) is well known to have improved analyte throughput, sensitivity, and chromatographic peak resolution compared with other analytical methods (Wang et al., 2015). The components and use of IMP and diazepam *in vitro* and *in vivo* were scientifically and systematically evaluated by UPLC-MS/MS to determine their safe clinical use.

## Material and Methods

### Chemicals and Reagents

IMP (purity > 98%) was purchased from MUST (Chengdu, China). Diazepam was purchased from Tianjin KingYork Pharmaceutical Co., Ltd. Clonazepam, nordazepam, and temazepam (purity > 98%) were purchased from Sigma-Aldrich (St.Louis, MO, USA), and clonazepam was used as the internal standard (IS). NADPH was purchased from Roche (Shanghai, China). Ultra-pure water was prepared by a Milli-Q puriﬁcation system (Millipore, Bedford, MA, USA). Analytical grade formic acid was provided by Sigma-Aldrich (St. Louis, MO, USA). UPLC grade acetonitrile and methanol were purchased from Merck Company (Darmstadt, Germany). The BCA Protein Assay Kit was purchased from Beyotime (Beijing, China). Blank rat plasma samples were supplied by drug-free rats from the Laboratory Animal Center of Wenzhou Medical University. All other chemicals for this study were reagent grade and used without further puriﬁcation.

### Instrumentation and Conditions

A UPLC-MS/MS system with ACQUITY I-Class UPLC and a XEVO TQD triple quadrupole mass spectrometer (Waters Corp., Milford, MA, USA), equipped with an electrospray ionization (ESI) interface, was used to analyze the compounds in positive mode. The UPLC system was comprised of a binary solvent manager and a sample manager with flow-through needle. Masslynx 4.1 software (Waters Corp.) was used for data acquisition and instrument control.

Diazepam, nordazepam, and temazepam were separated using a UPLC BEH C18 column (2.1 mm × 50 mm, 1.7 μm; Waters Corp.) maintained at 40^◦^C. The initial mobile phase consisted of acetonitrile and formic acid water (0.1% formic acid v/v) with gradient elution at a ﬂow rate of 0.4 mL/min and an injection volume of 2 μL. Elution was performed in a linear gradient where the acetonitrile content increased from 10% to 30% within 0.3 min and then continued to increase from 30% to 95% between 0.3 and 2.0 min. The acetonitrile content was maintained at 95% for 0.5 min, then dropped to 10% within 0.1 min. The total run time of the analytes was 3 min. After each injection, the sample manager underwent a needle wash process with methanol–water.

Nitrogen was used as the desolvation gas (1000 L/h) and cone gas (50 L/h). The ion monitoring voltage conditions were as follows: capillary voltage: 2.5 kV; source temperature: 150°C; desolvation temperature: 500°C. Multiple reaction monitoring methods were used for quantitative analysis. The transitions of diazepam, nordazepam, temazepam, and clonazepam are shown in **Table 1**.

**Table 1:** The transitions of diazepam, nordazepam, temazepam, and clonazepam.

Compound	Parent	Daughter	Cone (V)	Collision (eV)
Diazepam	285.1	193.1	35	30
Nordazepam	271.1	139.9	32	30
Temazepam	301.1	254.9	26	24
Clonazepam	316.1	269.9	38	24

### Preparation and Quantification of Protein Concentration of RLMs

In this study, rats were weighed and anesthetized with 10% chloral hydrate (0.35 mL/100g) followed by hepatic perfusion with ice-cold saline (0.9% NaCl), weighed, and homogenized. Subsequently, the homogenized liquid was centrifuged at 9000 rpm for 30 minutes at 4°C. The supernatant was then removed and centrifuged at 105,000 rpm for 60 minutes under the same conditions. The precipitate was reconstituted with 0.15 mol of a KCl-PBS buffer solution (pH 7.4) and dispensed. After repeating the above procedure, the precipitate was stored in a KCl-PBS buffer solution containing 0.25 mol/L sucrose at -80°C until use. The protein concentrations of RLMs were determined by BCA assay.

### Effects of IMP on the Metabolism of Diazepam *In Vitro*

Incubation mixtures were prepared in a total volume of 200 μL as follows: 0.44 mg/mL RLMs, 100 mM potassium phosphate buffer (pH 7.4), 50 μM diazepam, 1 mM NADPH, and IMP. For the IC_50_ (half maximal inhibitory concentration) determination, concentrations of 0.1, 0.5, 1, 2.5, 5, 10, 25, 50, and 100 µM IMP were used. For determination of the type of inhibition of IMP on diazepam metabolism, inhibition constant (Ki) values were obtained by incubating IMP (0, 0.5, 1, 2, and 4μM) with a series of concentrations of diazepam (12.5, 25, 50, and 100μM).The above preparations were mixed and incubated at 37°C for 5 min. NADPH was added to initiate a 30-min reaction process, which was then transferred to ice, and 200 μL acetonitrile was added to stop the reaction. Twenty microliters of clonazepam was added to the incubation system, vortexed for 2 min, and centrifuged at 13000 rpm for 5 min, and a 2-μL aliquot of the supernatant was used for UPLC-MS/MS analysis.

### Method Validation

**Figure 2:**
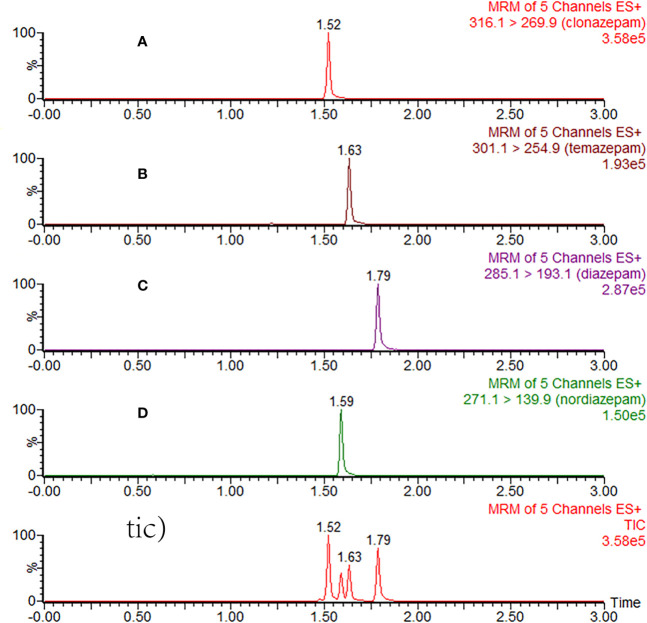
Typical MRM chromatograms of clonazepam **(A)**, temazepam **(B)**, diazepam **(C)**, nordazepam **(D)**, and total ion chromatography (tic) in plasma samples after 3 h of oral administration of diazepam.

The assay method was fully validated in terms of precision, accuracy, recovery, matrix effect, and stability, according to the guidelines set by the EMA (2011) (European Medicines Agency) and US FDA (FDA, 2018) (Food and Drug Administration) **Figure 2**. Quality control (QC) samples were prepared at low, medium, and high concentrations and used for the method validation in 6 replicates.

### Animal Experiments

Male Sprague-Dawley (SD) rats with body weights of 220-250 g were obtained from the Wenzhou Medical University Experimental Animal Center. The animals were kept in a controlled environment at 20–26°C and 55 ± 15% relative humidity under a 12-h light-dark cycle. Diet was prohibited for 12 h before the experiment, but water was freely available. All experimental procedures and protocols were reviewed and approved by the Animal Ethics Committee of Wenzhou Medical University, in line with the Guidelines for the Care and Use of Laboratory Animals.

### Effects of IMP the Pharmacokinetic of Diazepam In Vivo

Twelve SD male rats were randomly selected and assigned to either treatment group or control group. Treatment group rats were pretreated with an oral administration of IMP (50 mg/kg) daily for 2 weeks. Control group rats were pretreated with saline for 2 weeks. All rats were given diazepam (10 mg/kg) after the last administration of IMP. 300 μL blood was collected from the tail veins of both groups into centrifuge tubes with heparin according to a time series of 0.083, 0.25, 0.5, 1, 2, 3, 4, 6, 8, and 12h. Each blood sample was immediately centrifuged at 3,000 rpm for 10 min, and the supernatant was separated, transferred to another centrifuge tube, and stored at -20°C until analysis. Before analysis, the plasma sample was thawed to room temperature. An aliquot of 20 μL of the IS working solution (0.5μg/mL clonazepam) was added to 50 μL of plasma sample in a 1.5-mL centrifuge tube, followed by the addition of 200 μL of acetonitrile. The tubes were vortexed for 1 min. After centrifugation at 13000rpm for 5 min, the supernatant (2 μL) was injected into the UPLC-MS/MS system for analysis.

### Statistical Analyses

Non-compartmental analysis was used to calculate the pharmacokinetic parameters using Drug and Statistics software (v.3.2.8). The average plasma concentration–time curve was drawn according to the mean drug concentrations at each time point. The IC_50_ was calculated with GraphPad v.8 (GraphPad Software Inc., San Diego, CA, USA). Statistical analyses were evaluated by student T test (SPSS 24.0, IBM, Chicago, IL, USA). Differences were considered statistically significant when P < 0.05.

## Results

### Effects of IMP on the Metabolism of Diazepam In Vitro

**Figure 3:**
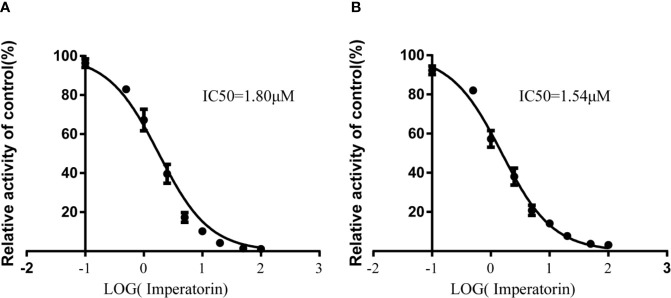
IMP with various concentrations for half-maximal inhibitory concentration (IC_50_) of diazepam to nordazepam **(A)** and temazepam **(B)** in RLMs (Mean ± SD, n=3).

**Figure 4:**
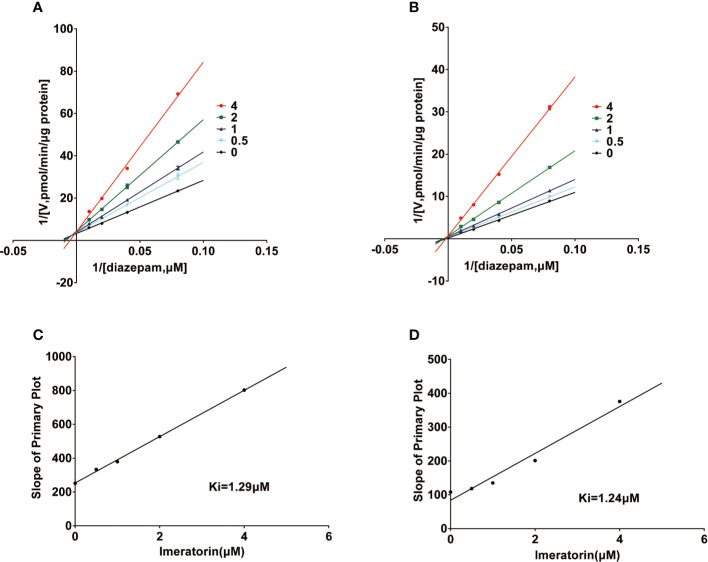
Lineweaver-Burk plot and the secondary plot for Ki in the inhibition of diazepam to nordazepam **(A, C)** and temazepam **(B, D)** by IMP with various concentrations RLMs (Mean ± SD, n=3).

Various concentrations of IMP ranging from 0.1 μM to 100 μM were used to determine the IC_50_ values of diazepam metabolism to temazepam and nordiazepam (**Figure 3**), which were found to be 1.80 μM and1.54 μM, respectively. Enzyme kinetic analyses *via* Lineweaver-Burk plots and secondary plots were used to fit the inhibition models of IMP on diazepam metabolism in RLMs. As shown in **Figure 4**, the types of inhibition by IMP on diazepam metabolism to temazepam and nordiazepam were all competitive. The Ki values were calculated as 1.24 μM and 1.29 μM, respectively.

### Method Validation

This method was in accordance with EMA and FDA regulatory requirements for bioanalytical methods. Details of the method validation results were illustrated in **Tables 2** and **3**.

**Table 2:** Precision, accuracy, recovery, and matrix effect of diazepam, nordazepam, and temazepam in rat plasma (n = 6).

Analyte	Nominal concentration (ng/mL)	intra-day		inter-day		Recovery (%)	Matrix (%)
		PrecisionRSD (%)	AccuracyRE (%)	PrecisionRSD (%)	AccuracyRE (%)		
diazepam	1.5	8.22	101.20	3.54	102.9	93.11	95.40
	15	9.03	98.29	8.12	102.06	86.72	94.58
	150	6.92	101.88	7.72	103.07	91.68	95.70
nordazepam	0.3	11.01	106.00	3.92	103.99	87.61	91.95
	3	6.54	101.50	8.12	103.67	85.28	98.75
	30	10.95	108.74	9.68	104.02	90.43	94.13
temazepam	0.3	6.36	98.33	10.3	97.04	86.11	95.09
	3	7.25	107.80	9.71	103.14	94.76	91.53
	30	6.74	108.70	9.77	106.72	86.31	89.62

**Table 3:** Stability of diazepam, nordazepam, and temazepam in rat plasma (n=6).

Analyte	Nominal concentration(ng/mL)	Short-term (room temperature, 24 h)		Short-term (4℃)		Freeze/thaw(-20°C to room temperature)		Long-term(-80℃, 2 weeks)	
		RSD(%)	RE(%)	RSD(%)	RE(%)	RSD(%)	RE(%)	RSD(%)	RE(%)
diazepam	1.5	4.35	105.04	5.16	97.38	2.79	103.04	6.44	103.92
	15	4.48	106.24	2.76	97.65	6.40	103.14	2.73	95.92
	150	5.33	105.12	4.72	103.49	4.01	104.66	6.08	104.71
nordazepam	0.3	8.75	104.95	3.16	103.77	4.23	102.21	8.04	104.20
	3	4.28	95.80	4.99	102.19	9.14	95.77	5.36	106.92
	30	6.85	108.45	8.26	108.79	7.81	106.33	10.90	105.97
temazepam	0.3	8.55	97.12	7.99	104.91	4.34	98.69	7.55	104.98
	3	9.78	103.78	7.28	106.87	5.47	104.13	3.40	103.69
	30	5.92	102.95	9.32	104.66	8.15	105.72	7.01	104.62

### Effects of IMP on the Pharmacokinetic of Diazepam In Vivo

**Figure 5:**
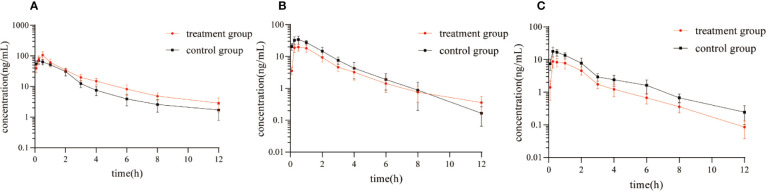
Mean plasma concentration-time curve of **(A)** diazepam, **(B)** nordazepam, and **(C)** temazepam in treatment group and control group after oral administration of diazepam (Mean ± SD, n = 6).

**Figure 5** showed the mean plasma concentration-time curve of the treatment group or control group after oral administration of diazepam. The pharmacokinetic parameters of diazepam were analyzed using a non-compartment model, as shown in **Table 4**. After oral administration, diazepam was rapidly absorbed in the plasma and reached a maximum concentration of 77.05 ± 12.25 ng/mL at 0.5 hours. When pre-administered with IMP for 14 days, the AUC_(0-12h)_ of diazepam significantly increased from 157.25 ± 19.52 to 219.77 ± 41.91 μg/h/L (*P*<0.05). The C_max_ of diazepam significantly increased from 77.05 ± 12.25 to 107.11 ± 30.73 ng/mL (*P* < 0.05), and the clearance (CLz/F) was obviously decreased from 59.16 ± 7.14 to 42.38 ± 6.57 L/h/kg (*P* < 0.05). Although the MRT_(0-12)_ of diazepam increased from 3.71 ± 1.7 to 4.36 ± 2.12 h, the difference was not significant (*P* > 0.05). The t_1/2_ of diazepam in treatment group rats was shorter compared with control group rats (4.97 ± 2.16 vs. 4.89 ± 3.48 h, respectively), but the difference was not significant (*P* > 0.05).

**Table 4:** The main pharmacokinetic parameters of diazepam in treatment group and control group (*n* = 6, mean ± SD).

Pharmacokinetics parameters	treatment group	control group
AUC_(0-12h)_ (μg/h/L)	219.77 ± 41.91*	157.25 ± 19.52
AUC_(0-∞)_ (μg/h/L)	241.37 ± 42.43*	171.04 ± 20.04
MRT_(0-t)_ (h)	2.21 ± 0.37	2.21 ± 0.26
MRT_(0-∞)_ (h)	4.36 ± 2.12	3.71 ± 1.7
t_1/2_z (h)	4.89 ± 3.48	4.97 ± 2.16
T_max_ (h)	0.5 ± 0*	0.26 ± 0.13
Vz/F (L/kg)	200.19 ± 42.05*	427.67 ± 198.54
CLz/F(L/h/kg)	42.38 ± 6.57*	59.16 ± 7.14
C_max_ (μg/L)	107.11 ± 30.73*	77.05 ± 12.25

*P < 0.05 indicates significant differences from the control.

AUC, area under the plasma concentration–time curve; CL, plasma clearance;

C_max_, maximum plasma concentration; MRT, mean residence time;

SD, standard deviation; t_1/2_, half-life; T_max_, maximum plasma time.

The results of the correlational analysis of nordazepam are displayed in **Table 5**. After pretreatment with IMP, the AUC_(0-12h)_ and C_max_ of nordazepam were significantly decreased from 35.22 ± 8.65 to 23.09 ± 4.11 ng/mL and from 78.55 ± 12.07 to 50.15 ± 9.96 μg/h/L, respectively (*P* < 0.05). Vz/F and CLz/F in the treatment group increased 2.42- and 1.55-fold, respectively, compared with the treatment group (*P* < 0.05). Although the MRT_(0-12)_ and T_max_ of nordazepam increased from 1.94 ± 0.36 to 2.23 ± 0.31 h and from 0.46 ± 0.1 to 0.63 ± 0.31, respectively, the difference was not significant (*P* > 0.05). The results obtained from the preliminary analysis of temazepam are compared in **Table 6**. After pretreatment with IMP, the AUC_(0-12h)_ and C_max_ of temazepam significantly decreased from 18.47 ± 5.45 to 9.96 ± 2.3 ng/mL and from 41.17 ± 6.52 to 21.73 ± 3.73 μg/h/L, respectively (*P* < 0.05). Vz/F and CLz/F in the treatment group increased 1.73- and 1.86-fold, respectively, compared with the control group (*P* < 0.05). The MRT_(0-12)_ and T_max_ of nordazepam were not significantly different between the treatment group or control group (*P* > 0.05).

**Table 5:** The main pharmacokinetic parameters of nordazepam in treatment group and control group (*n* = 6, mean ± SD).

Pharmacokinetics parameters	treatment group	control group
AUC_(0-12h)_ (μg/h/L)	50.15 ± 9.96*	78.55 ± 12.07
AUC_(0-∞)_ (μg/h/L)	51.49 ± 10.07*	79.2 ± 12.86
MRT_(0-t)_ (h)	2.23 ± 0.31	1.94 ± 0.36
MRT_(0-∞)_ (h)	2.62 ± 0.44	2.04 ± 0.42
t_1/2_z (h)	2.91 ± 1.25*	1.59 ± 0.72
T_max_ (h)	0.63 ± 0.31	0.46 ± 0.1
Vz/F (L/kg)	696.1 ± 226.04*	286.66 ± 103.02
CLz/F(L/h/kg)	201.14 ± 42.84*	128.96 ± 20.13
C_max_ (μg/L)	23.09 ± 4.11*	35.22 ± 8.65

*P < 0.05 indicate significant differences from the control.

**Table 6:** The main pharmacokinetic parameters of temazepam in treatment group and control group (*n* = 6, mean ± SD).

Pharmacokinetics parameters	treatment group	control group
AUC_(0-12h)_ (μg/h/L)	21.73 ± 3.73*	41.17 ± 6.52
AUC_(0-∞)_ (μg/h/L)	22.58 ± 3.21*	42.3 ± 6.33
MRT_(0-t)_ (h)	2.22 ± 0.29	2.28 ± 0.26
MRT_(0-∞)_ (h)	3.08 ± 1.46	2.69 ± 0.55
t_1/2_z (h)	2.14 ± 0.48	2.6 ± 1.21
T_max_ (h)	0.46 ± 0.29	0.54 ± 0.37
Vz/F (L/kg)	1304.05 ± 284.46*	753.65 ± 300.88
CLz/F (L/h/kg)	449.83 ± 58.7*	241.83 ± 43.74
C_max_ (μg/L)	9.96 ± 2.3*	18.47 ± 5.45

*P < 0.05 indicate significant differences from the control.

## Discussion

Pharmacokinetic interactions between food and drugs may occur during absorption, distribution, metabolism, or excretion. DDIs generally occur through their pharmacokinetic properties, with metabolic drugs having the highest incidence of DDIs (Qiang and Kexin, 2015; Liu et al., 2018). Furocoumarins are a group of natural products that widely present in fruits, beverages, and herbs in different forms (Hung et al., 2017). Therefore, many components of furocoumarins influence the activities of P450, P-gp, organic cation/carnitine transporter, and organic anion transporter (Wang et al., 2012; Dong et al., 2018), which may cause drug interactions.

Previous studies have used liquid-high-resolution mass spectrometry to detect dozens of metabolites, mainly oxidation products, in the urine of rats orally administered IMP (Qiao et al., 2013). This indicates that IMP can undergo extensive metabolism in rats by CYP1A2, CYP2B6, CYP2D6, CYP2C19, CYP2C9, and CYP3A4 (Kang et al., 2011). The drug may take time to diffuse from plasma to other organs and tissues. According to previous reports, the absolute bioavailability of IMP is more than 40%. (Chen et al., 2015).

Because diazepam can rapidly enter the cranial nerve after intravenous injection and cause mild central nervous system inhibition, it is widely used in psychiatric practice. The metabolism of diazepam is intricate. It is first demethylated by CYP3A4 and CYP2C19 in the liver to nordiazepam, and hydroxylated by CYP3A4 to form the active metabolite temazepam. Both nordiazepam and temazepam are further metabolized to oxazepam. Then, the combination of temazepam and oxazepam by glucuronidation and glucuronic acid is largely eliminated. However, they are still biologically active and able to accumulate with continuous application (St-Pierre et al., 1993).

In vitro studies have shown that IMP has a strong inhibitory effect on diazepam metabolism in RLMs, and the IC_50_ values of temazepam and nordazepam were less than 1.8 μM, consistent with *in vivo* pharmacokinetic studies. The Ki values for temazepam and nordiazepam were very close, with values of 1.24 μM and 1.29 μM, respectively, indicating that IMP might inhibit diazepam in a competitive manner. IMP and diazepam might bind to the same active center of the enzyme. Conversely, IMP displayed a mixed type of inhibition on human CYP3A4 and CYP2C9 in reported studies (Kimura et al., 2010), which was different from our results, due to the species differences in CYP enzyme in metabolism.

In the *in vivo* study, when diazepam was combined with IMP, the values of AUC_(0-12h)_, AUC_(0-∞)_ and Cmax of diazepam significantly increased, while CLz/F decreased about 1.4 fold, compared to the control group. For pharmacokinetic parameters of the metabolites of diazepam, nordiazepam, and temazepam, IMP reduced the AUC_(0-12h)_, AUC_(0-∞),_ and Cmax but increased the CL/F. The results of the *in vivo* experiment revealed that IMP had an influence on the metabolism of diazepam, which causes drug-drug interactions (DDIs). Like the IMP, another furocoumarin, psoralen, is a mechanism-based inactivator of CYP2B6 (Ji et al., 2015). In addition, psoralen has been demonstrated to significantly increase exposure of anastrozole, a P450 substrate, in rat vivo, and significantly decrease intrinsic clearance rates by psoralen pre-treatment in rat liver microsome(Zhang et al., 2018), indicating that furocoumarins result in DDI.

## Conclusion

The purpose of the current study was to explore the effect of IMP on diazepam *in vitro* and *in vivo*. These findings have significant implications for the understanding of this effect. *In* *vitro*, IMP to nordazepam and temazepam has a lower IC_50_ value, indicating that IMP might inhibit the metabolism of diazepam in RLMs. The mechanism of IMP in RLMs, represented by a Lineweaver-Burk Plot, indicates that IMP might inhibit diazepam in a competitive manner. When orally administered, IMP can increase the AUC of diazepam, prolong its t_1/2_, and decrease its CL *in vivo*, which has a risk of inhibiting interactions with CYP enzymes. Our study provides guidance for the clinical treatment of diazepam for anxiety and insomnia in combination with food or herbs containing IMP, and these data still require further confirmation by conducting clinical trials in the future.

## Data Availability Statement

The raw data supporting the conclusions of this article will be made available by the authors, without undue reservation.

## Ethics Statement

The animal study was reviewed and approved by Animal Ethics Committee of Wenzhou Medical University.

## Author Contributions

WQ, QZ, and YZ contributed to the conception and design of the study. YZ, DM, ZW, BW, SW, PG, and FC performed experiments. DD performed the statistical analysis. YZ and DM wrote the first draft of the manuscript. ZW, SW, QZ, and DD wrote sections of the manuscript. FC revised the manuscript. All authors contributed to the article and approved the submitted version. WQ is accountable for all aspects of the work in ensuring that questions related to the accuracy or integrity of any part of the work are appropriately investigated and resolved.

## Funding

This work was supported by grants funded by the Medical and Health Research Project of Zhejiang province (2017KY731), Natural Science Foundation of Zhejiang and Zhejiang Pharmaceutical Association Joint Foundation (LYY18H280003), City-level public welfare technology application research project of Lishui (2016GYX32 & 2017GYX15), and CAMS Innovation Fund for Medical Sciences (2018-I2M-1-002).

## Conflict of Interest

The authors declare that the research was conducted in the absence of any commercial or financial relationships that could be construed as a potential conflict of interest.
